# Supplementary material for: How the water-soluble hemicarcerand incarcerates guests at room temperature decoded with modular simulations
Source: Commun Chem. 2021 Mar 1;4:26. doi: 10.1038/s42004-021-00469-3 (PMC9814894; doi:10.1038/s42004-021-00469-3)

Supplementary Information

How the Water-Soluble Hemicarcerand Incarcerates Guests at Room Temperature Decoded with Modular Simulations

Katherine G. McFerrin and Yuan-Ping Pang*

Computer-Aided Molecular Design Laboratory, Mayo Clinic, Rochester, MN, USA

*E-mail: camdl1@icloud.com

Fig. S1 ……………………………………………………………………Pages 2–13

Fig. S2 ……………………………………………………………………Page 14

Fig. S3 ……………………………………………………………………Page 15

Fig. S4 ……………………………………………………………………Page 16

Fig. S5 ……………………………………………………………………Page 17

Fig. S1. Time series of radius of gyration of the Octacid4 cavity for all 20 distinct and independent MD simulations at 298 K, 340 K, and 363 K.

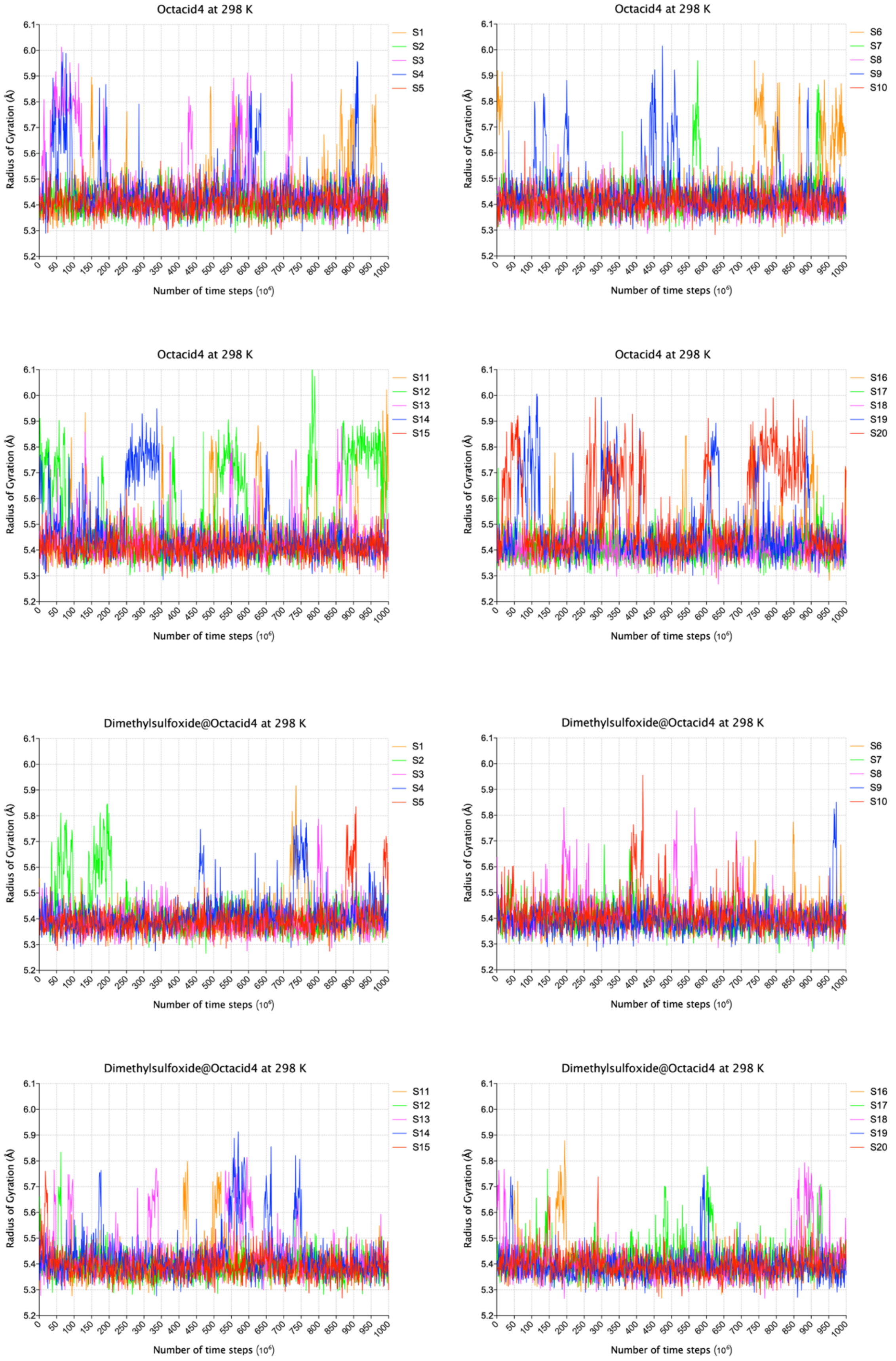

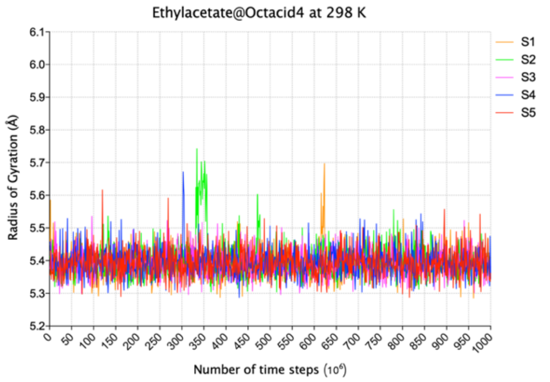
Ethylacetate@Octacid4 at 298 K
S1
S2
S3
S4
S5
Radius of Gyration (Å)
Number of time steps (10^6)

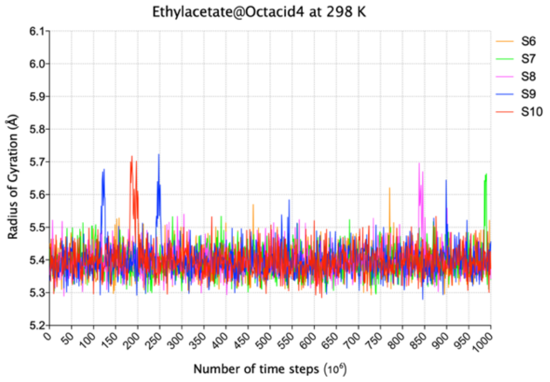
Ethylacetate@Octacid4 at 298 K
S6
S7
S8
S9
S10
Radius of Gyration (Å)
Number of time steps (10^6)

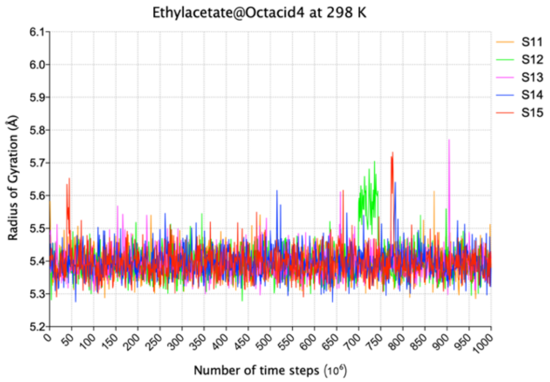
Ethylacetate@Octacid4 at 298 K
S11
S12
S13
S14
S15
Radius of Gyration (Å)
Number of time steps (10^6)

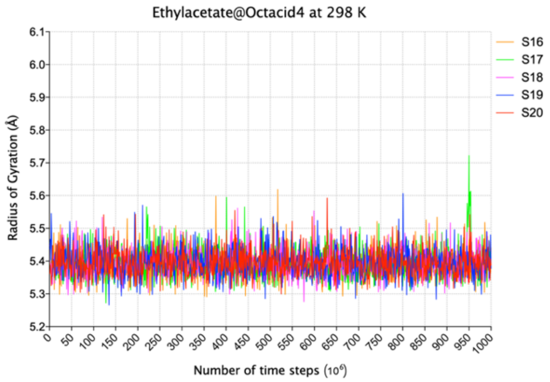
Ethylacetate@Octacid4 at 298 K
S16
S17
S18
S19
S20
Radius of Gyration (Å)
Number of time steps (10^6)

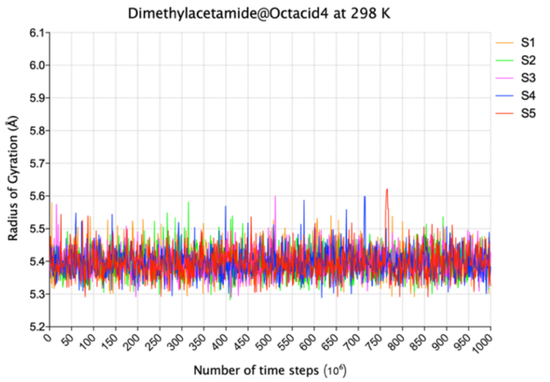
Dimethylacetamide@Octacid4 at 298 K
S1
S2
S3
S4
S5
Radius of Gyration (Å)
Number of time steps (10^6)

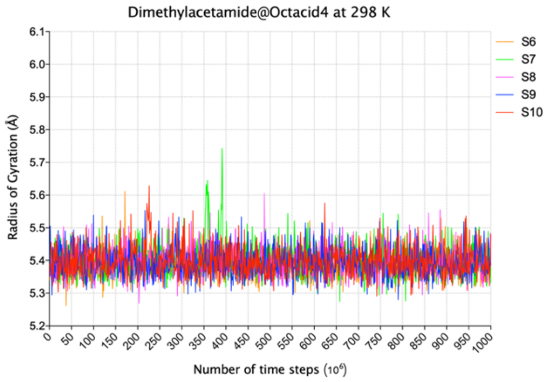
Dimethylacetamide@Octacid4 at 298 K
S6
S7
S8
S9
S10
Radius of Gyration (Å)
Number of time steps (10^6)

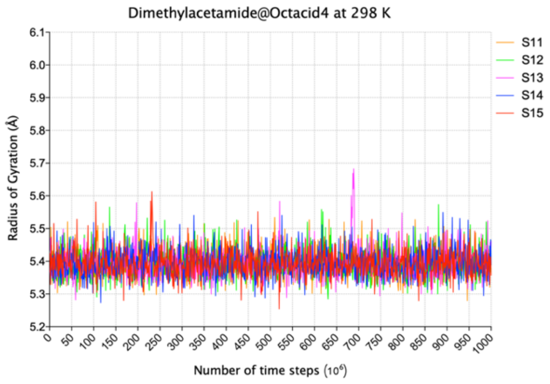
Dimethylacetamide@Octacid4 at 298 K
S11
S12
S13
S14
S15
Radius of Gyration (Å)
Number of time steps (10^6)

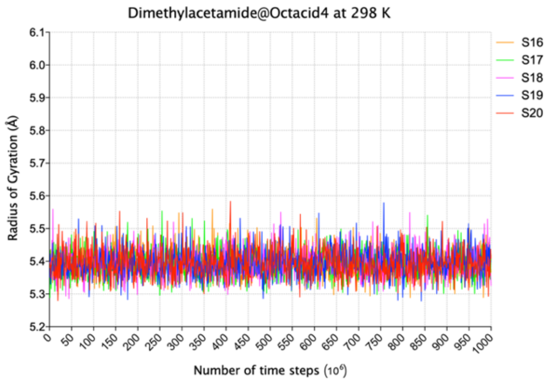
Dimethylacetamide@Octacid4 at 298 K
S16
S17
S18
S19
S20
Radius of Gyration (Å)
Number of time steps (10^6)

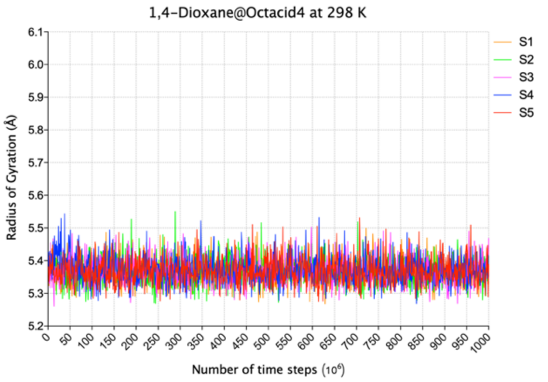
1,4-Dioxane@Octacid4 at 298 K
Radius of Gyration (Å)
Number of time steps (10⁶)
S1
S2
S3
S4
S5

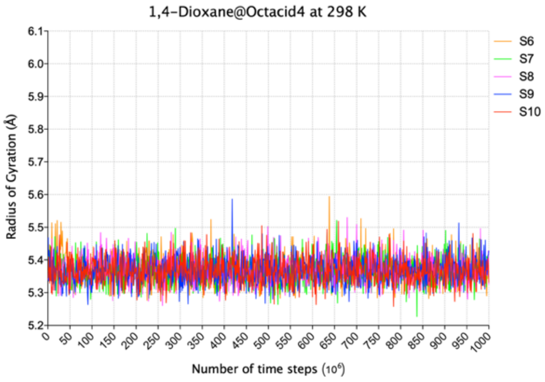
1,4-Dioxane@Octacid4 at 298 K
Radius of Gyration (Å)
Number of time steps (10⁶)
S6
S7
S8
S9
S10

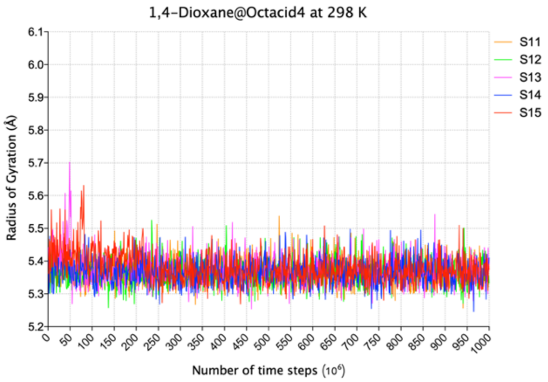
1,4-Dioxane@Octacid4 at 298 K
Radius of Gyration (Å)
Number of time steps (10⁶)
S11
S12
S13
S14
S15

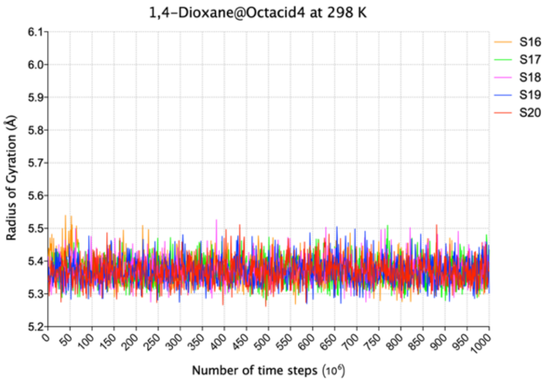
1,4-Dioxane@Octacid4 at 298 K
Radius of Gyration (Å)
Number of time steps (10⁶)
S16
S17
S18
S19
S20

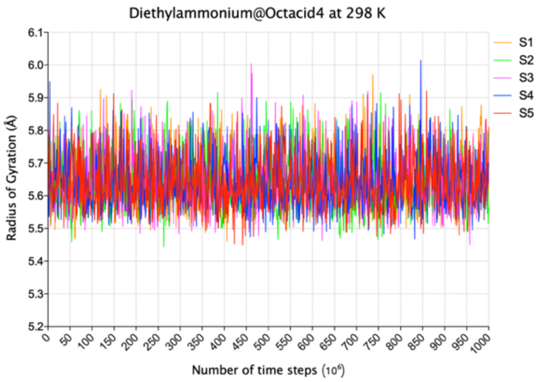
Diethylammonium@Octacid4 at 298 K
Radius of Gyration (Å)
Number of time steps (10⁶)
S1
S2
S3
S4
S5

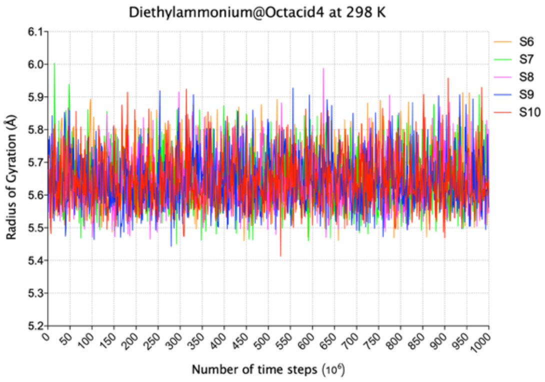
Diethylammonium@Octacid4 at 298 K
Radius of Gyration (Å)
Number of time steps (10⁶)
S6
S7
S8
S9
S10

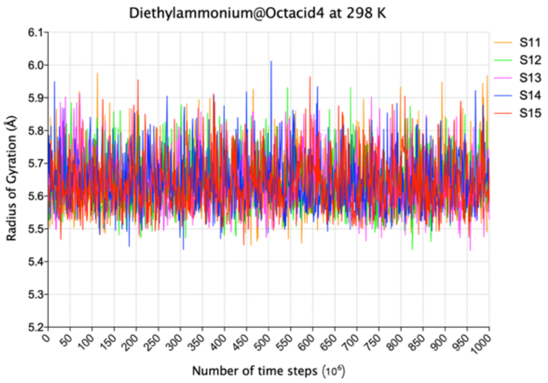
Diethylammonium@Octacid4 at 298 K
Radius of Gyration (Å)
Number of time steps (10⁶)
S11
S12
S13
S14
S15

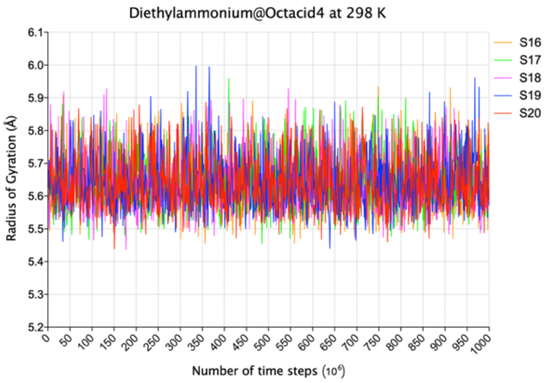
Diethylammonium@Octacid4 at 298 K
Radius of Gyration (Å)
Number of time steps (10⁶)
S16
S17
S18
S19
S20

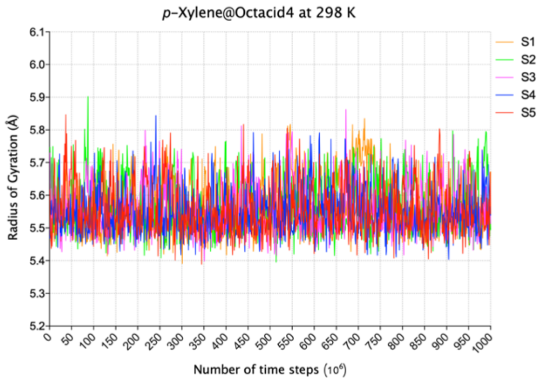
p-Xylene@Octacid4 at 298 K
Radius of Gyration (Å)
Number of time steps (10⁶)
S1
S2
S3
S4
S5

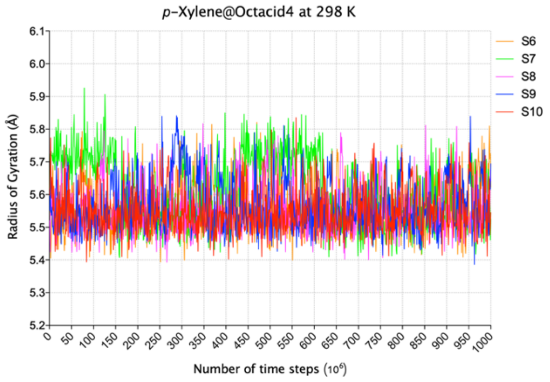
p-Xylene@Octacid4 at 298 K
Radius of Gyration (Å)
Number of time steps (10⁶)
S6
S7
S8
S9
S10

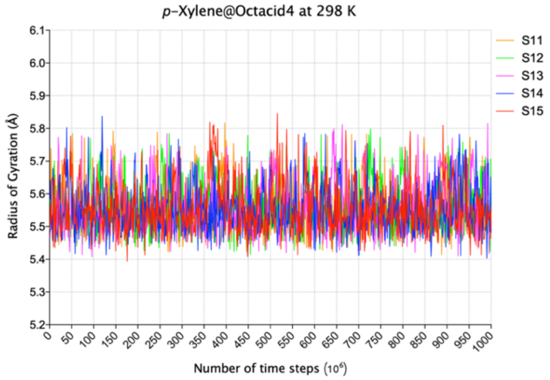
p-Xylene@Octacid4 at 298 K
Radius of Gyration (Å)
Number of time steps (10⁶)
S11
S12
S13
S14
S15

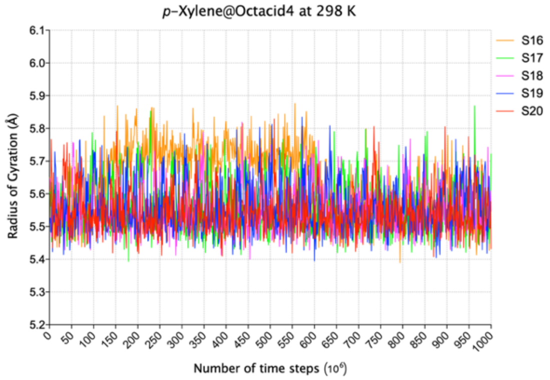
p-Xylene@Octacid4 at 298 K
Radius of Gyration (Å)
Number of time steps (10⁶)
S16
S17
S18
S19
S20

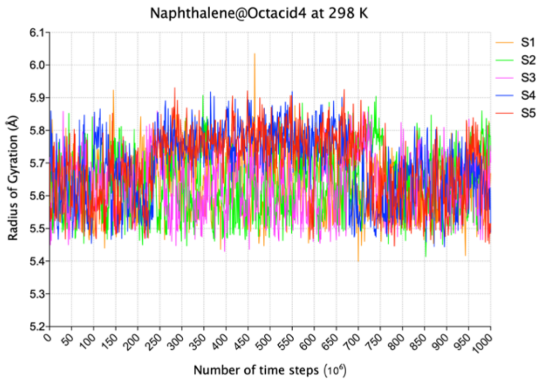
Naphthalene@Octacid4 at 298 K
Radius of Gyration (Å)
Number of time steps (10⁶)
S1
S2
S3
S4
S5

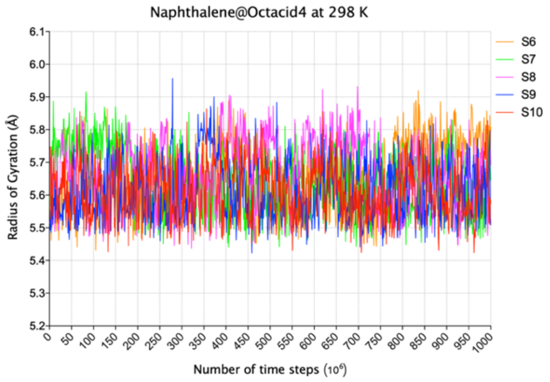
Naphthalene@Octacid4 at 298 K
Radius of Gyration (Å)
Number of time steps (10⁶)
S6
S7
S8
S9
S10

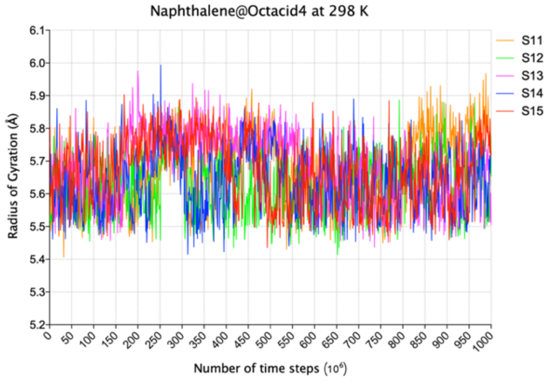
Naphthalene@Octacid4 at 298 K
Radius of Gyration (Å)
Number of time steps (10⁶)
S11
S12
S13
S14
S15

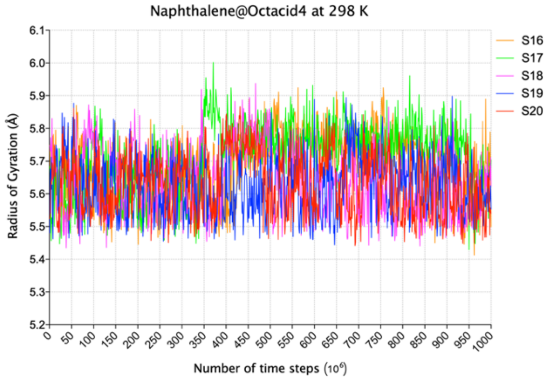
Naphthalene@Octacid4 at 298 K
Radius of Gyration (Å)
Number of time steps (10⁶)
S16
S17
S18
S19
S20

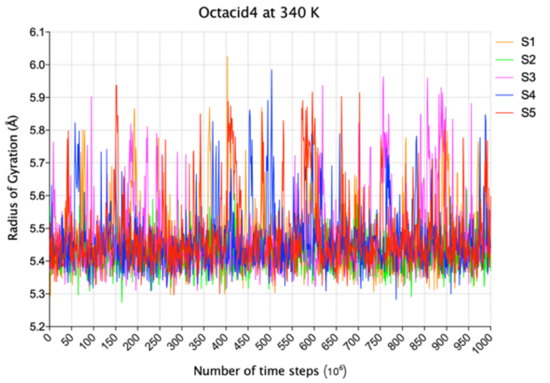
Octacid4 at 340 K
Radius of Gyration (Å)
Number of time steps (10⁶)
S1
S2
S3
S4
S5

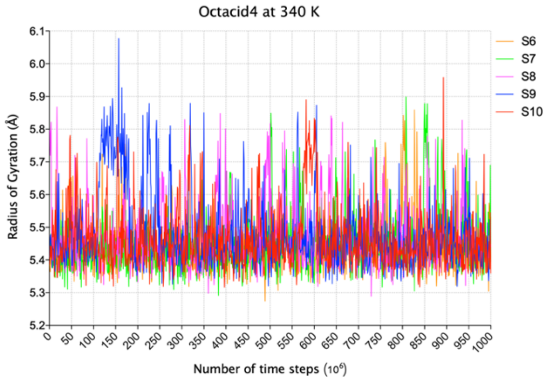
Octacid4 at 340 K
Radius of Gyration (Å)
Number of time steps (10⁶)
S6
S7
S8
S9
S10

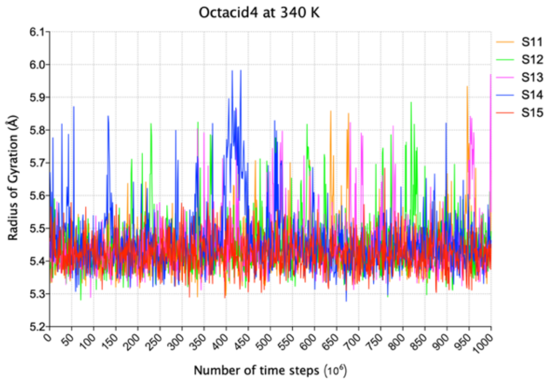
Octacid4 at 340 K
Radius of Gyration (Å)
Number of time steps (10⁶)
S11
S12
S13
S14
S15

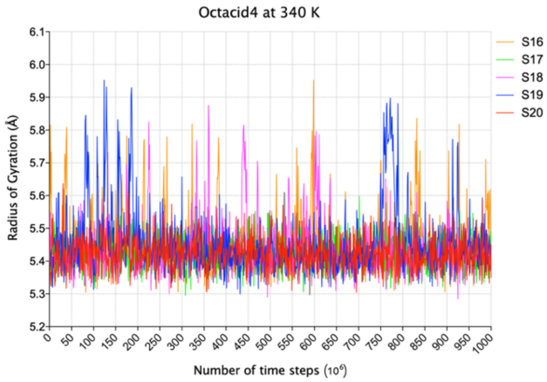
Octacid4 at 340 K
Radius of Gyration (Å)
Number of time steps (10⁶)
S16
S17
S18
S19
S20

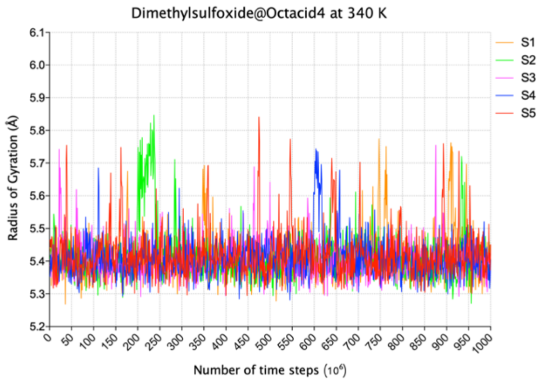
Dimethylsulfoxide@Octacid4 at 340 K
Radius of Gyration (Å)
Number of time steps (10⁶)
S1
S2
S3
S4
S5

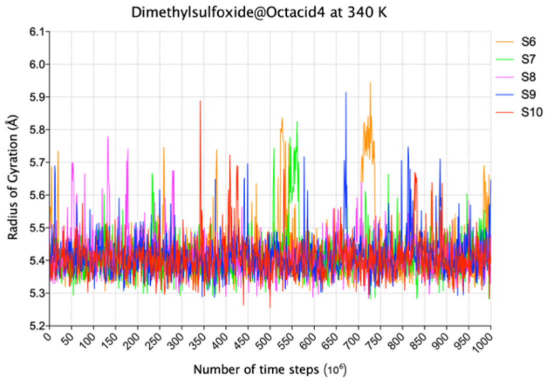
Dimethylsulfoxide@Octacid4 at 340 K
Radius of Gyration (Å)
Number of time steps (10⁶)
S6
S7
S8
S9
S10

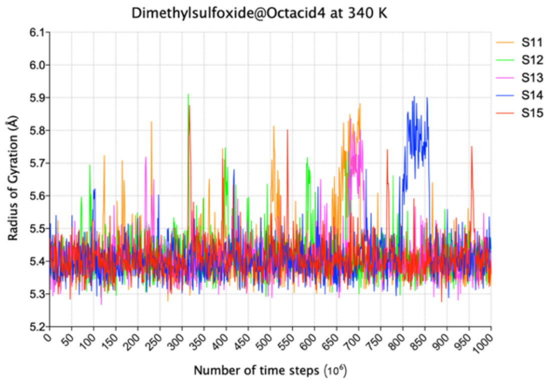
Dimethylsulfoxide@Octacid4 at 340 K
Radius of Gyration (Å)
Number of time steps (10⁶)
S11
S12
S13
S14
S15

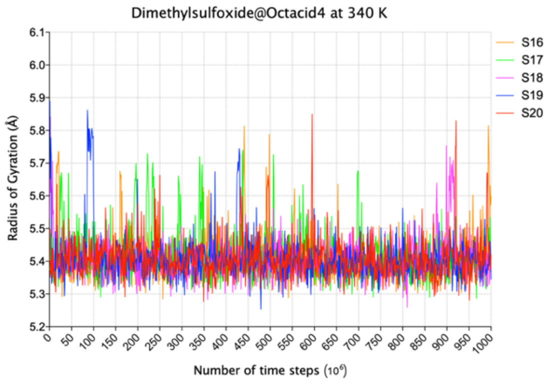
Dimethylsulfoxide@Octacid4 at 340 K
Radius of Gyration (Å)
Number of time steps (10⁶)
S16
S17
S18
S19
S20

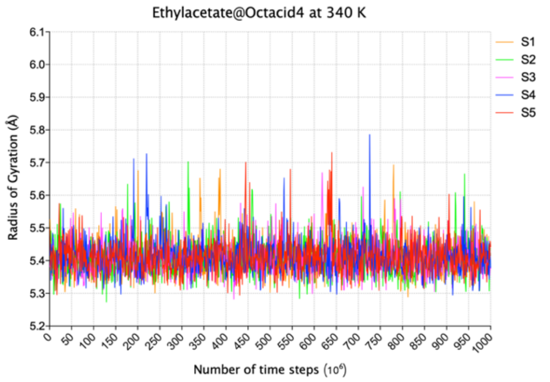
Ethylacetate@Octacid4 at 340 K
Radius of Gyration (Å)
Number of time steps (10⁶)
S1
S2
S3
S4
S5

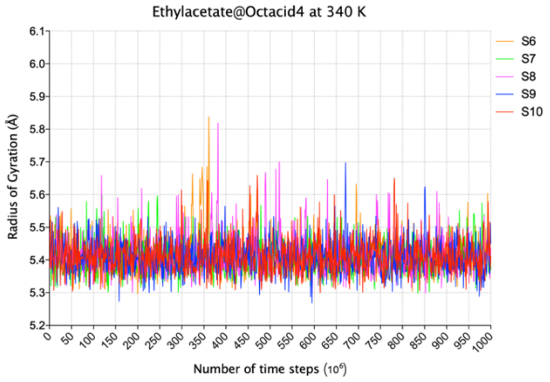
Ethylacetate@Octacid4 at 340 K
Radius of Gyration (Å)
Number of time steps (10⁶)
S6
S7
S8
S9
S10

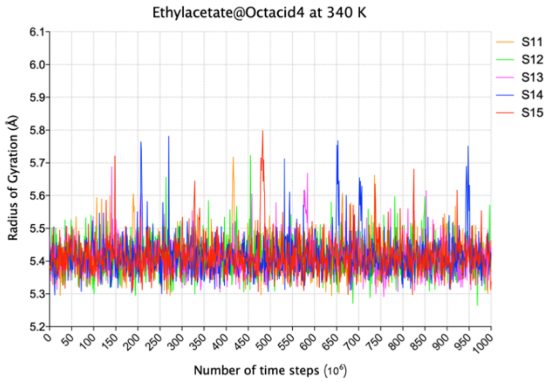
Ethylacetate@Octacid4 at 340 K
Radius of Gyration (Å)
Number of time steps (10⁶)
S11
S12
S13
S14
S15

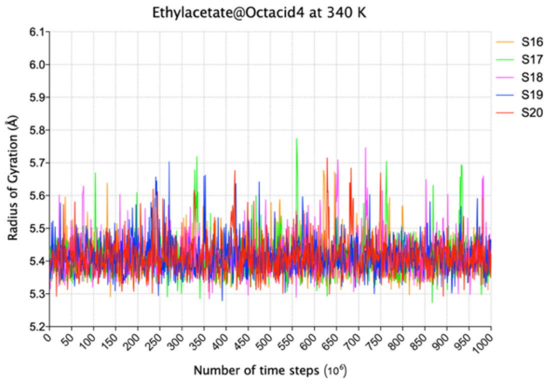
Ethylacetate@Octacid4 at 340 K
Radius of Gyration (Å)
Number of time steps (10⁶)
S16
S17
S18
S19
S20

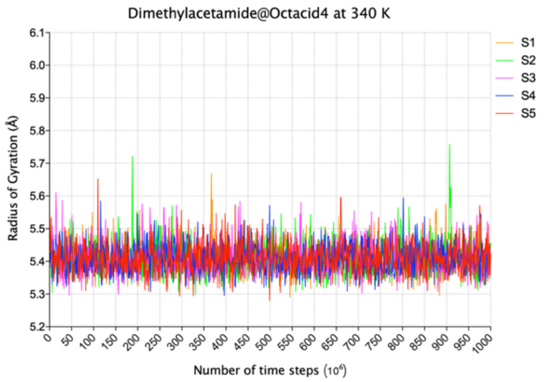
Dimethylacetamide@Octacid4 at 340 K
Radius of Gyration (Å)
Number of time steps (10⁶)
S1
S2
S3
S4
S5

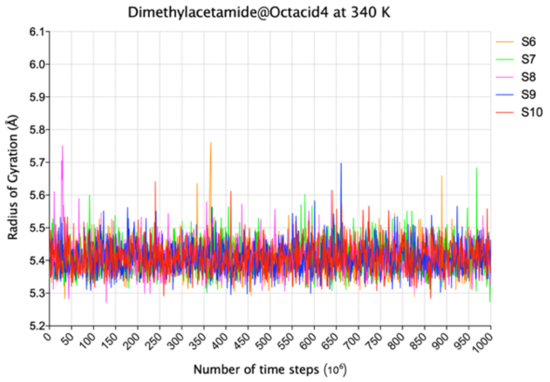
Dimethylacetamide@Octacid4 at 340 K
Radius of Gyration (Å)
Number of time steps (10⁶)
S6
S7
S8
S9
S10

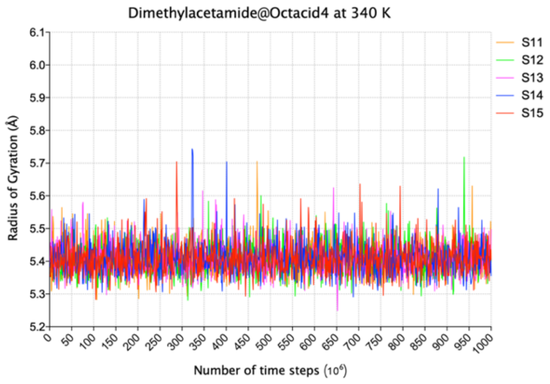
Dimethylacetamide@Octacid4 at 340 K
Radius of Gyration (Å)
Number of time steps (10⁶)
S11
S12
S13
S14
S15

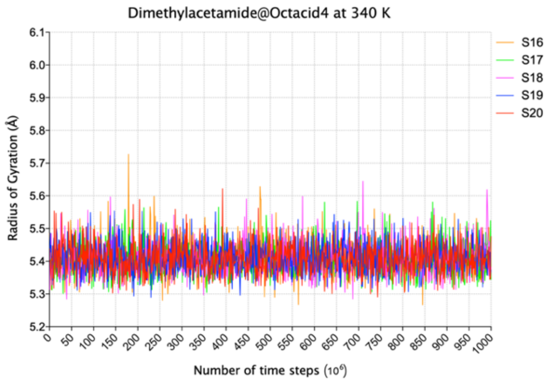
Dimethylacetamide@Octacid4 at 340 K
Radius of Gyration (Å)
Number of time steps (10⁶)
S16
S17
S18
S19
S20

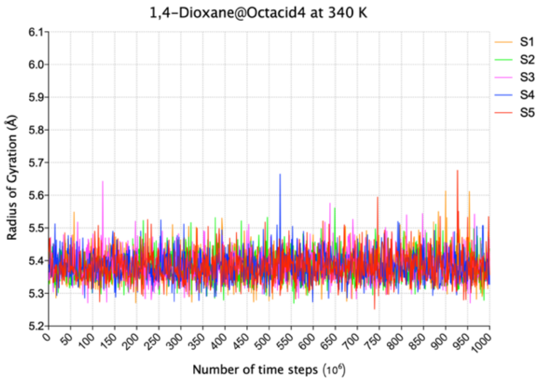
1,4-Dioxane@Octacid4 at 340 K
Radius of Gyration (Å)
Number of time steps (10^6)
S1
S2
S3
S4
S5

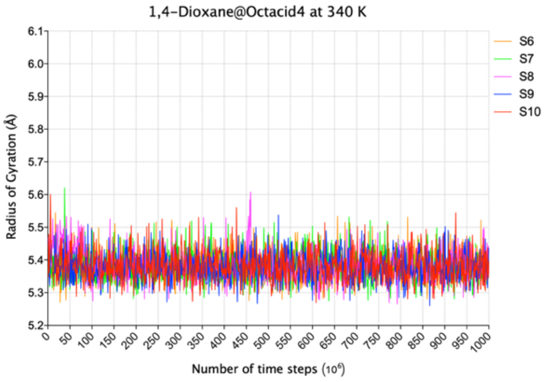
1,4-Dioxane@Octacid4 at 340 K
Radius of Gyration (Å)
Number of time steps (10^6)
S6
S7
S8
S9
S10

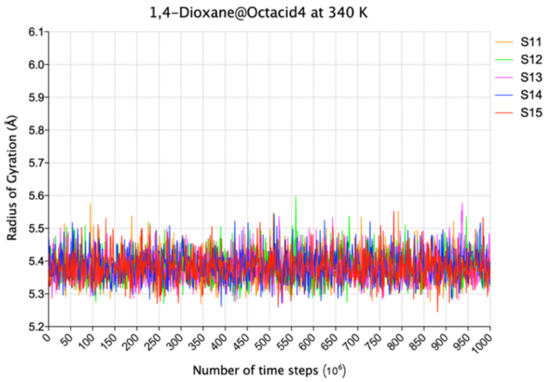
1,4-Dioxane@Octacid4 at 340 K
Radius of Gyration (Å)
Number of time steps (10^6)
S11
S12
S13
S14
S15

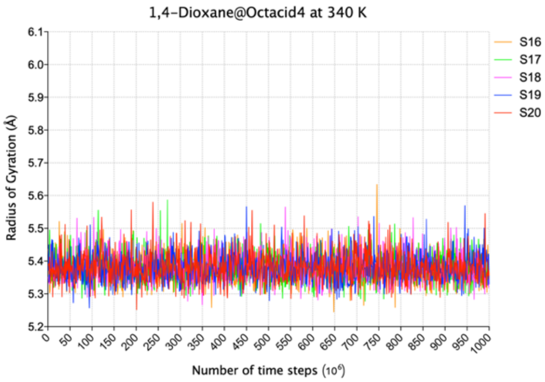
1,4-Dioxane@Octacid4 at 340 K
Radius of Gyration (Å)
Number of time steps (10^6)
S16
S17
S18
S19
S20

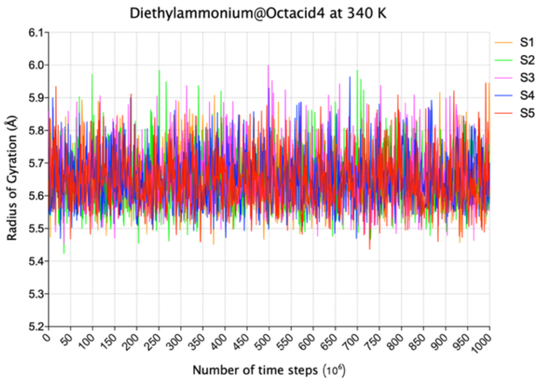
Diethylammonium@Octacid4 at 340 K
Radius of Gyration (Å)
Number of time steps (10^6)
S1
S2
S3
S4
S5

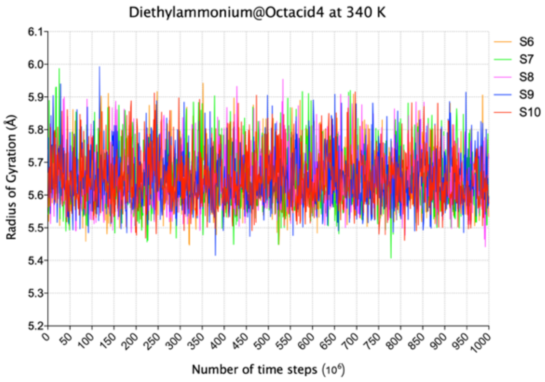
Diethylammonium@Octacid4 at 340 K
Radius of Gyration (Å)
Number of time steps (10^6)
S6
S7
S8
S9
S10

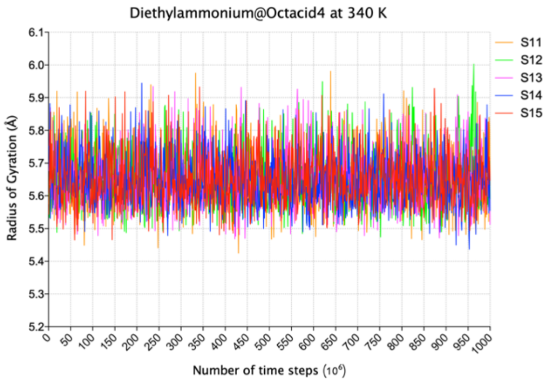
Diethylammonium@Octacid4 at 340 K
Radius of Gyration (Å)
Number of time steps (10^6)
S11
S12
S13
S14
S15

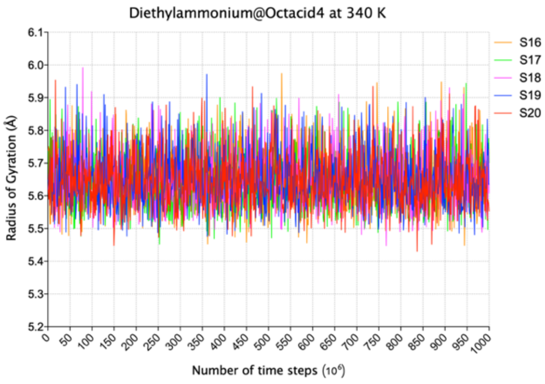
Diethylammonium@Octacid4 at 340 K
Radius of Gyration (Å)
Number of time steps (10^6)
S16
S17
S18
S19
S20

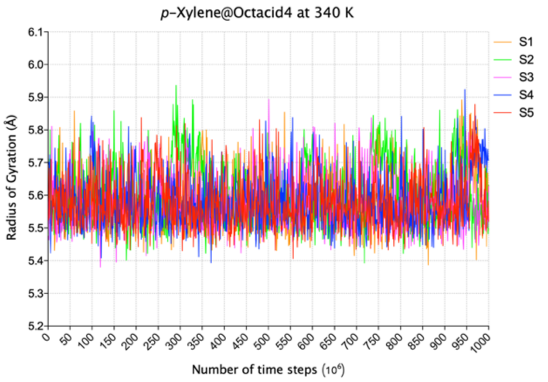
p-Xylene@Octacid4 at 340 K
Radius of Gyration (Å)
Number of time steps (10^6)
S1
S2
S3
S4
S5

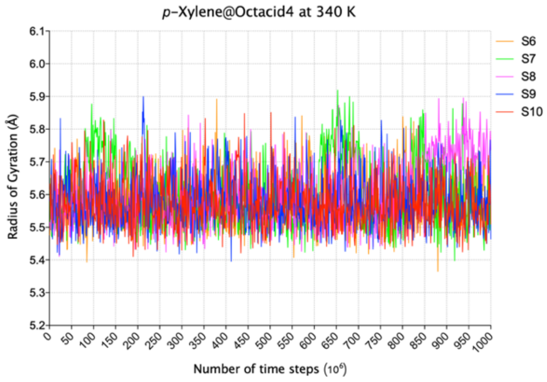
p-Xylene@Octacid4 at 340 K
Radius of Gyration (Å)
Number of time steps (10^6)
S6
S7
S8
S9
S10

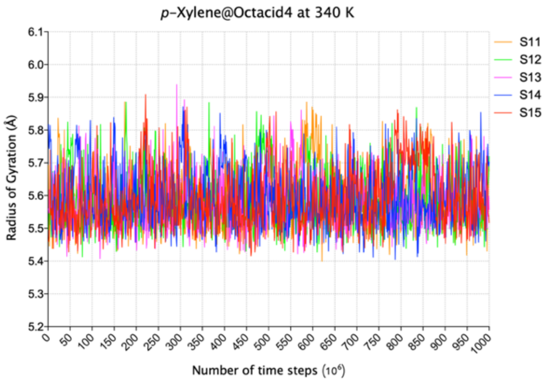
p-Xylene@Octacid4 at 340 K
Radius of Gyration (Å)
Number of time steps (10^6)
S11
S12
S13
S14
S15

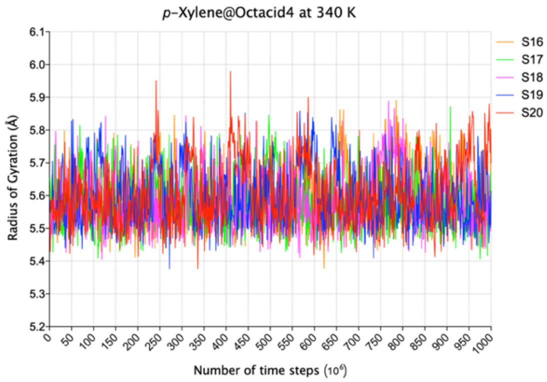
p-Xylene@Octacid4 at 340 K
Radius of Gyration (Å)
Number of time steps (10^6)
S16
S17
S18
S19
S20

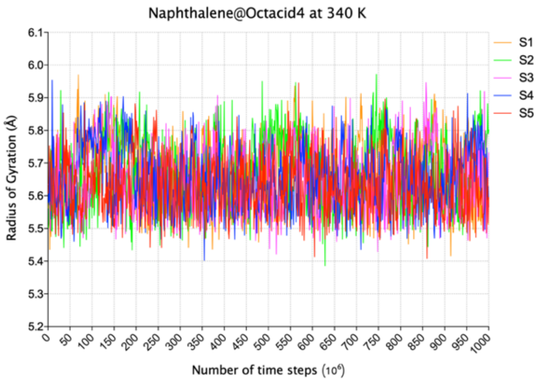
Naphthalene@Octacid4 at 340 K
Radius of Gyration (Å)
Number of time steps (10^6)
S1
S2
S3
S4
S5

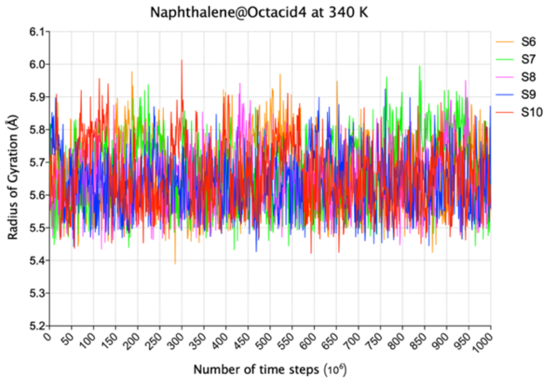
Naphthalene@Octacid4 at 340 K
Radius of Gyration (Å)
Number of time steps (10^6)
S6
S7
S8
S9
S10

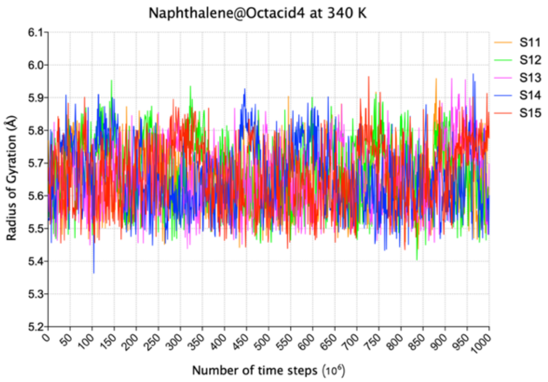
Naphthalene@Octacid4 at 340 K
Radius of Gyration (Å)
Number of time steps (10^6)
S11
S12
S13
S14
S15

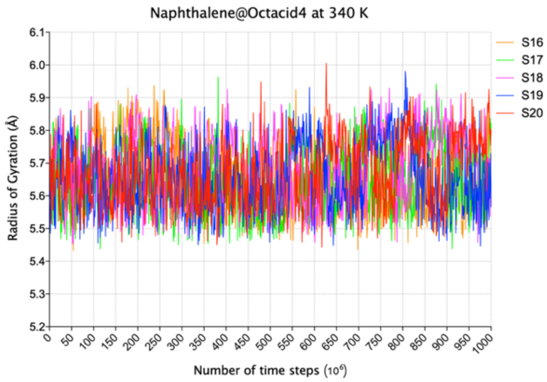
Naphthalene@Octacid4 at 340 K
Radius of Gyration (Å)
Number of time steps (10^6)
S16
S17
S18
S19
S20

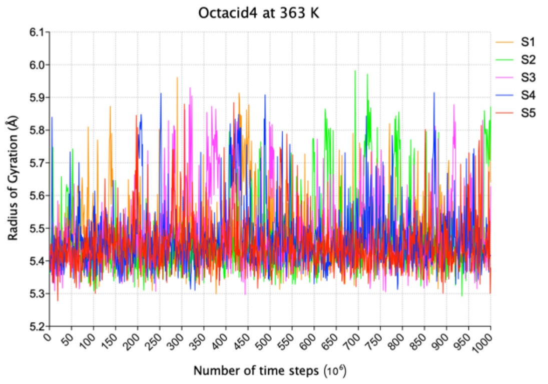
Octacid4 at 363 K
Radius of Gyration (Å)
Number of time steps (10⁶)
S1
S2
S3
S4
S5

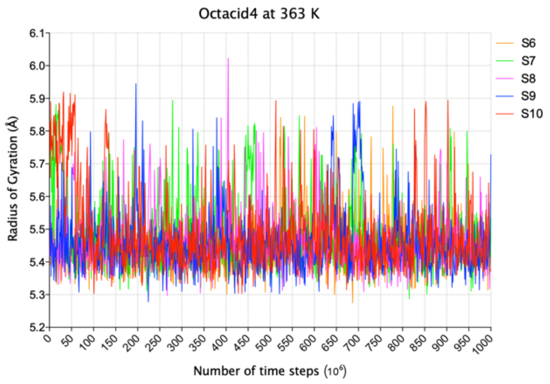
Octacid4 at 363 K
Radius of Gyration (Å)
Number of time steps (10⁶)
S6
S7
S8
S9
S10

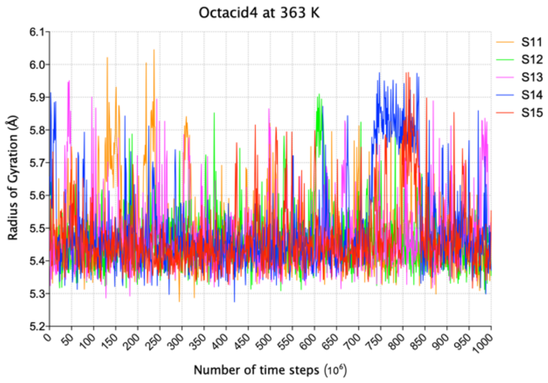
Octacid4 at 363 K
Radius of Gyration (Å)
Number of time steps (10⁶)
S11
S12
S13
S14
S15

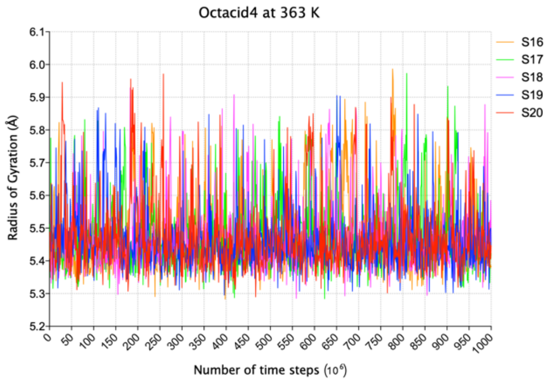
Octacid4 at 363 K
Radius of Gyration (Å)
Number of time steps (10⁶)
S16
S17
S18
S19
S20

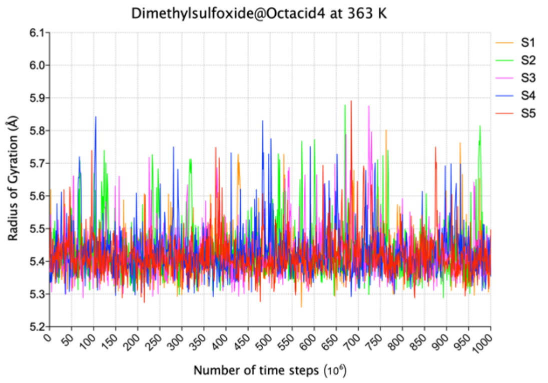
Dimethylsulfoxide@Octacid4 at 363 K
Radius of Gyration (Å)
Number of time steps (10⁶)
S1
S2
S3
S4
S5

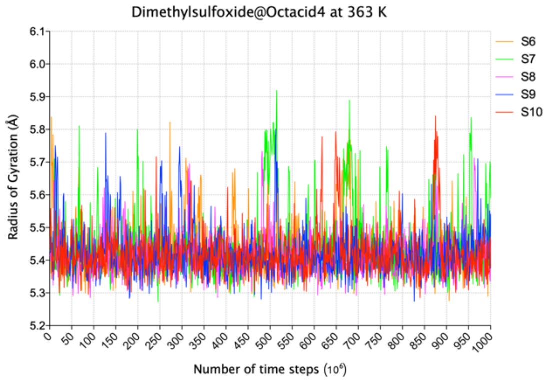
Dimethylsulfoxide@Octacid4 at 363 K
Radius of Gyration (Å)
Number of time steps (10⁶)
S6
S7
S8
S9
S10

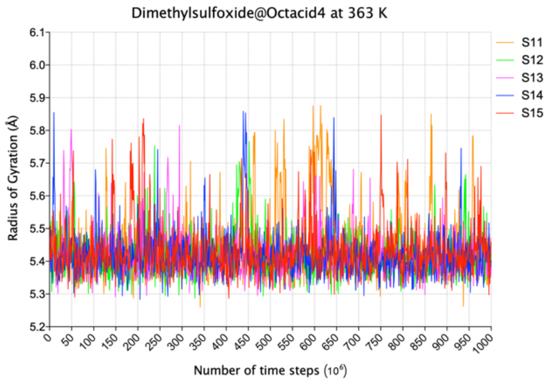
Dimethylsulfoxide@Octacid4 at 363 K
Radius of Gyration (Å)
Number of time steps (10⁶)
S11
S12
S13
S14
S15

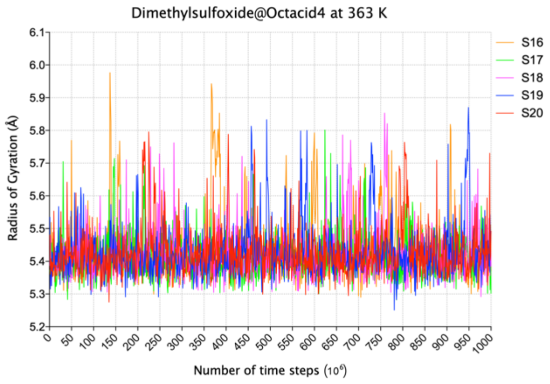
Dimethylsulfoxide@Octacid4 at 363 K
Radius of Gyration (Å)
Number of time steps (10⁶)
S16
S17
S18
S19
S20

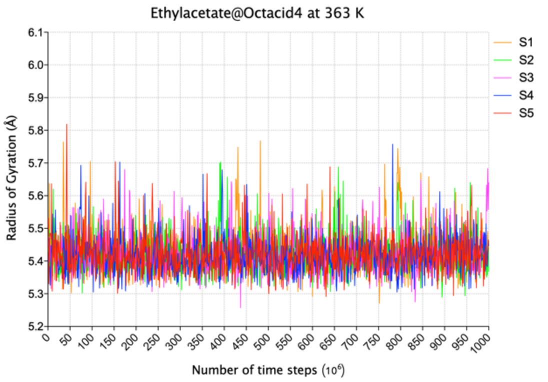
Ethylacetate@Octacid4 at 363 K
Radius of Gyration (Å)
Number of time steps (10^6)
S1
S2
S3
S4
S5

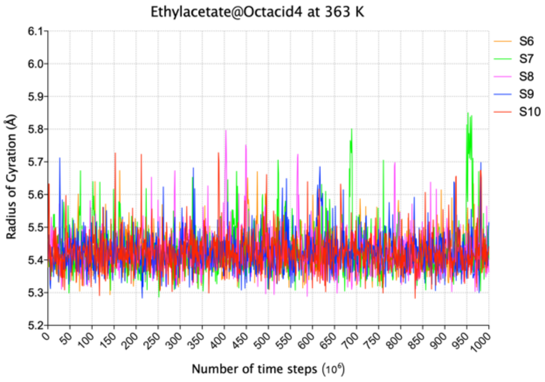
Ethylacetate@Octacid4 at 363 K
Radius of Gyration (Å)
Number of time steps (10^6)
S6
S7
S8
S9
S10

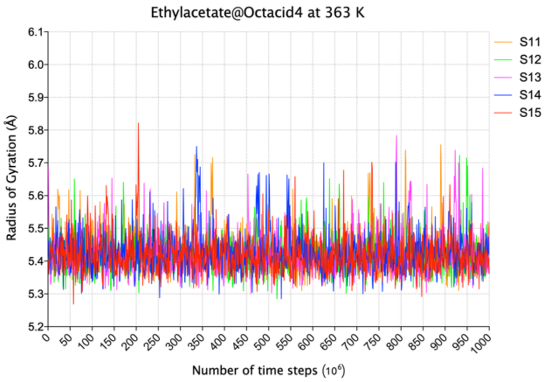
Ethylacetate@Octacid4 at 363 K
Radius of Gyration (Å)
Number of time steps (10^6)
S11
S12
S13
S14
S15

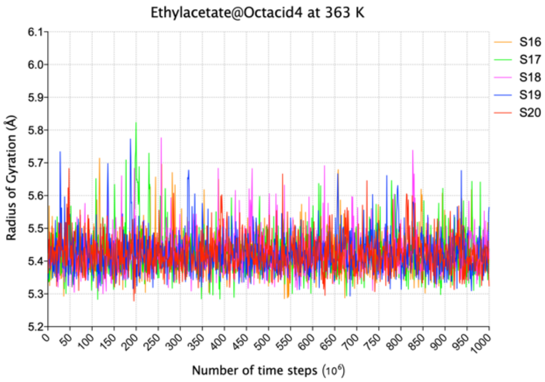
Ethylacetate@Octacid4 at 363 K
Radius of Gyration (Å)
Number of time steps (10^6)
S16
S17
S18
S19
S20

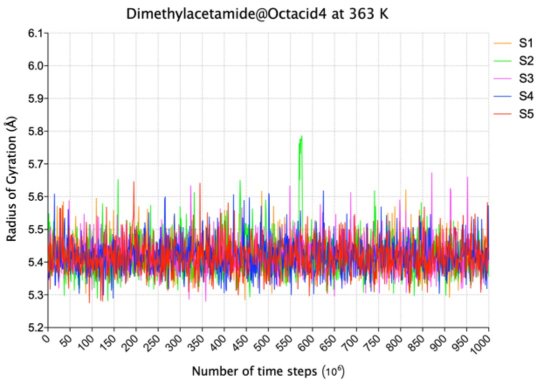
Dimethylacetamide@Octacid4 at 363 K
Radius of Gyration (Å)
Number of time steps (10^6)
S1
S2
S3
S4
S5

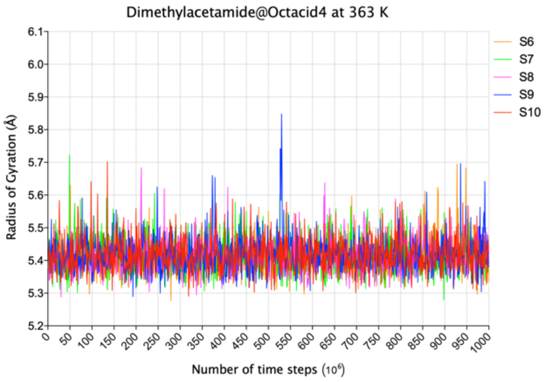
Dimethylacetamide@Octacid4 at 363 K
Radius of Gyration (Å)
Number of time steps (10^6)
S6
S7
S8
S9
S10

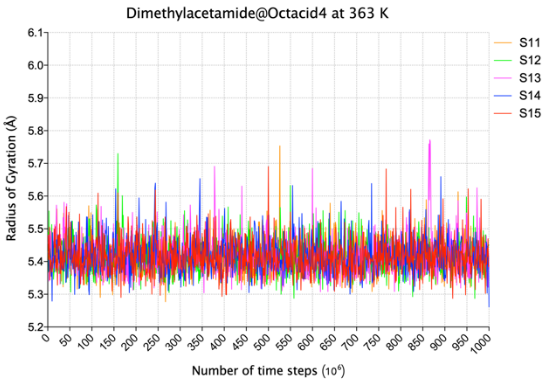
Dimethylacetamide@Octacid4 at 363 K
Radius of Gyration (Å)
Number of time steps (10^6)
S11
S12
S13
S14
S15

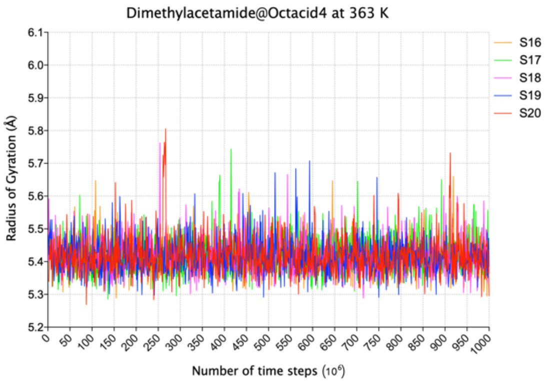
Dimethylacetamide@Octacid4 at 363 K
Radius of Gyration (Å)
Number of time steps (10^6)
S16
S17
S18
S19
S20

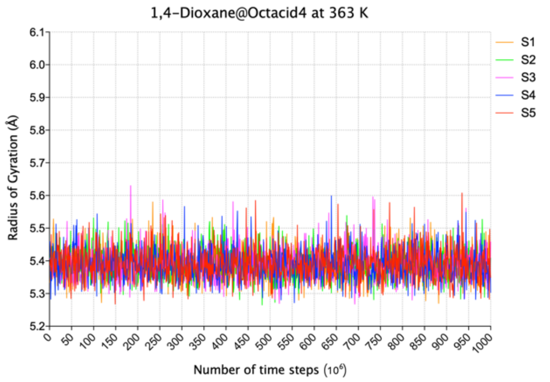
1,4-Dioxane@Octacid4 at 363 K
Radius of Gyration (Å)
Number of time steps (10^6)
S1
S2
S3
S4
S5

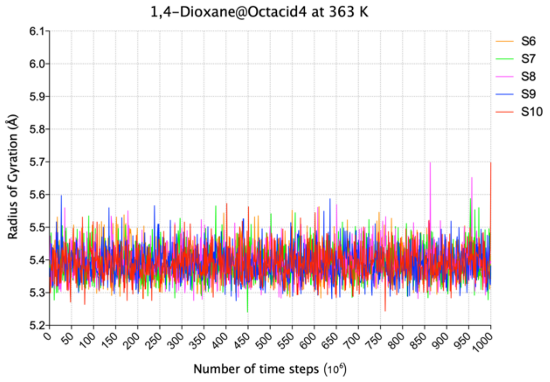
1,4-Dioxane@Octacid4 at 363 K
Radius of Gyration (Å)
Number of time steps (10^6)
S6
S7
S8
S9
S10

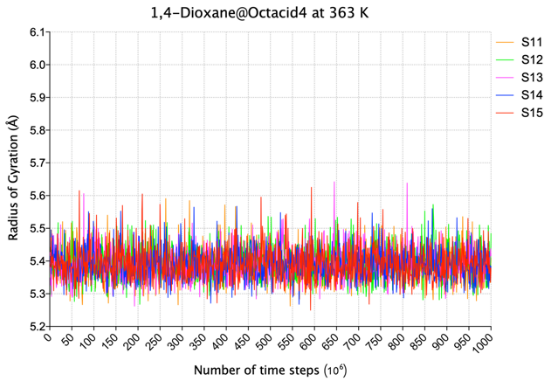
1,4-Dioxane@Octacid4 at 363 K
Radius of Gyration (Å)
Number of time steps (10^6)
S11
S12
S13
S14
S15

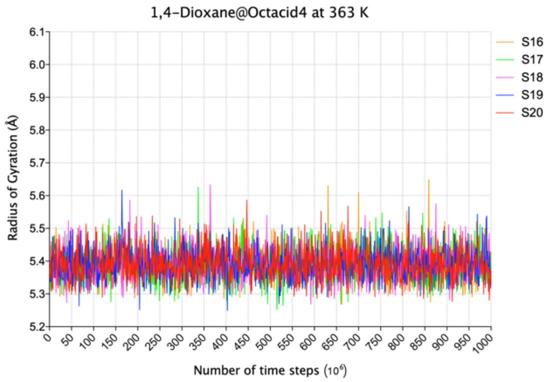
1,4-Dioxane@Octacid4 at 363 K
Radius of Gyration (Å)
Number of time steps (10^6)
S16
S17
S18
S19
S20

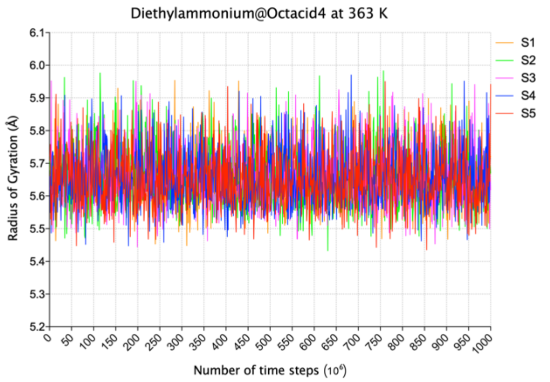
Diethylammonium@Octacid4 at 363 K
Radius of Gyration (Å)
Number of time steps (10^6)
S1
S2
S3
S4
S5

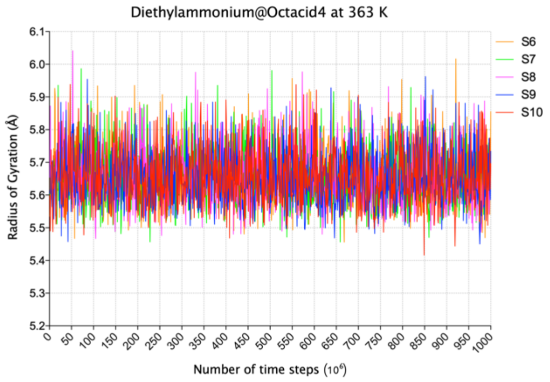
Diethylammonium@Octacid4 at 363 K
Radius of Gyration (Å)
Number of time steps (10^6)
S6
S7
S8
S9
S10

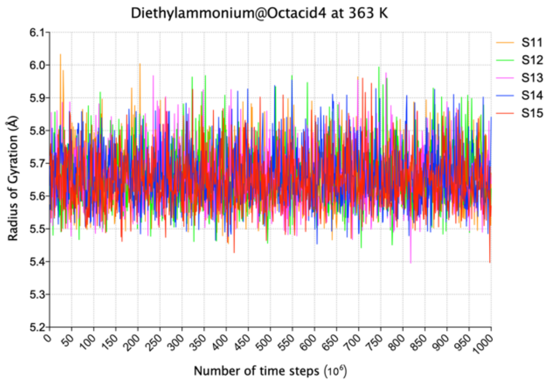
Diethylammonium@Octacid4 at 363 K
Radius of Gyration (Å)
Number of time steps (10^6)
S11
S12
S13
S14
S15

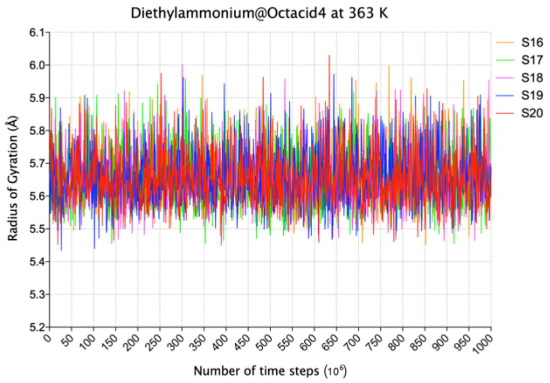
Diethylammonium@Octacid4 at 363 K
Radius of Gyration (Å)
Number of time steps (10^6)
S16
S17
S18
S19
S20

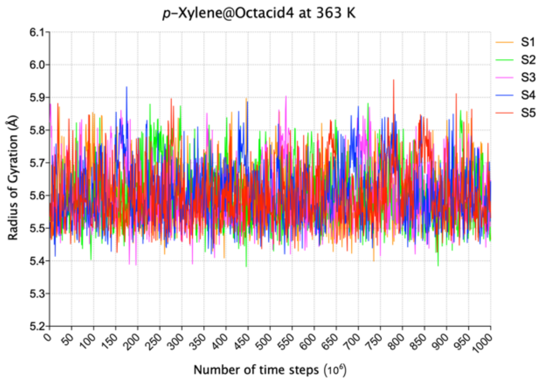
p-Xylene@Octacid4 at 363 K
Radius of Gyration (Å)
Number of time steps (10^6)
S1
S2
S3
S4
S5

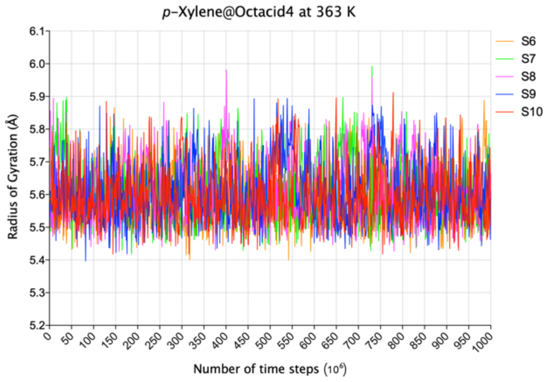
p-Xylene@Octacid4 at 363 K
Radius of Gyration (Å)
Number of time steps (10^6)
S6
S7
S8
S9
S10

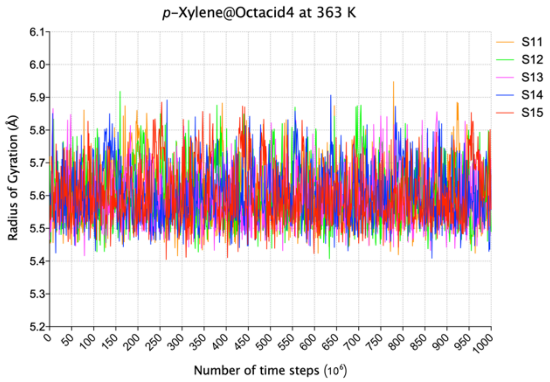
p-Xylene@Octacid4 at 363 K
Radius of Gyration (Å)
Number of time steps (10^6)
S11
S12
S13
S14
S15

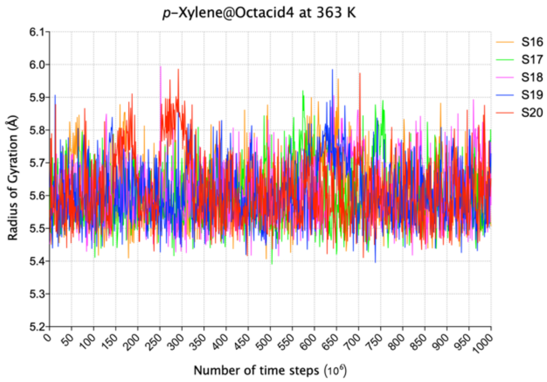
p-Xylene@Octacid4 at 363 K
Radius of Gyration (Å)
Number of time steps (10^6)
S16
S17
S18
S19
S20

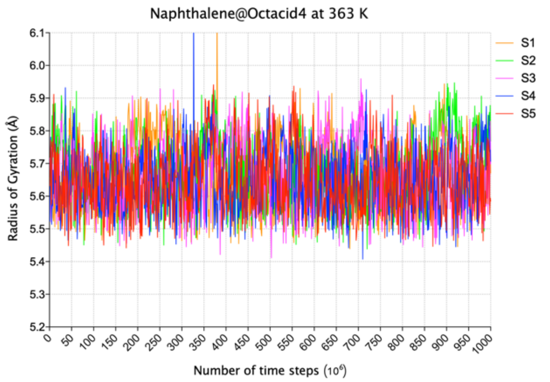
Naphthalene@Octacid4 at 363 K
Radius of Gyration (Å)
Number of time steps (10^6)
S1
S2
S3
S4
S5

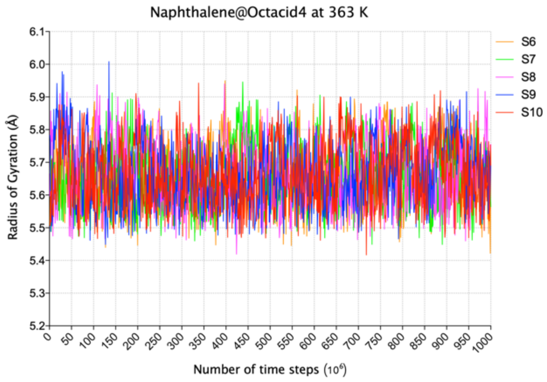
Naphthalene@Octacid4 at 363 K
Radius of Gyration (Å)
Number of time steps (10^6)
S6
S7
S8
S9
S10

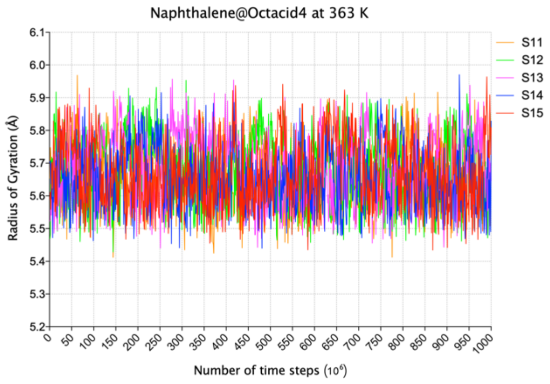
Naphthalene@Octacid4 at 363 K
Radius of Gyration (Å)
Number of time steps (10^6)
S11
S12
S13
S14
S15

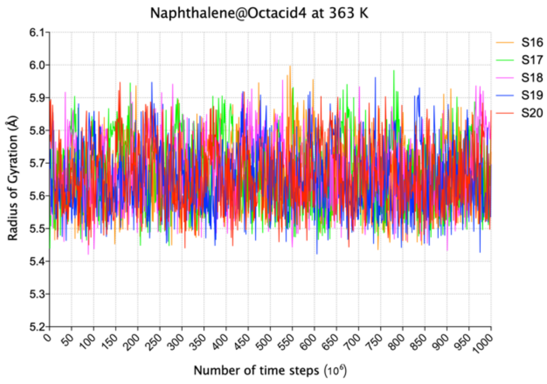
Naphthalene@Octacid4 at 363 K
Radius of Gyration (Å)
Number of time steps (10^6)
S16
S17
S18
S19
S20

Fig. S2. Time series of radius of gyration of the Octacid4 cavity for the first of 20 distinct and independent MD simulations at 340 K.

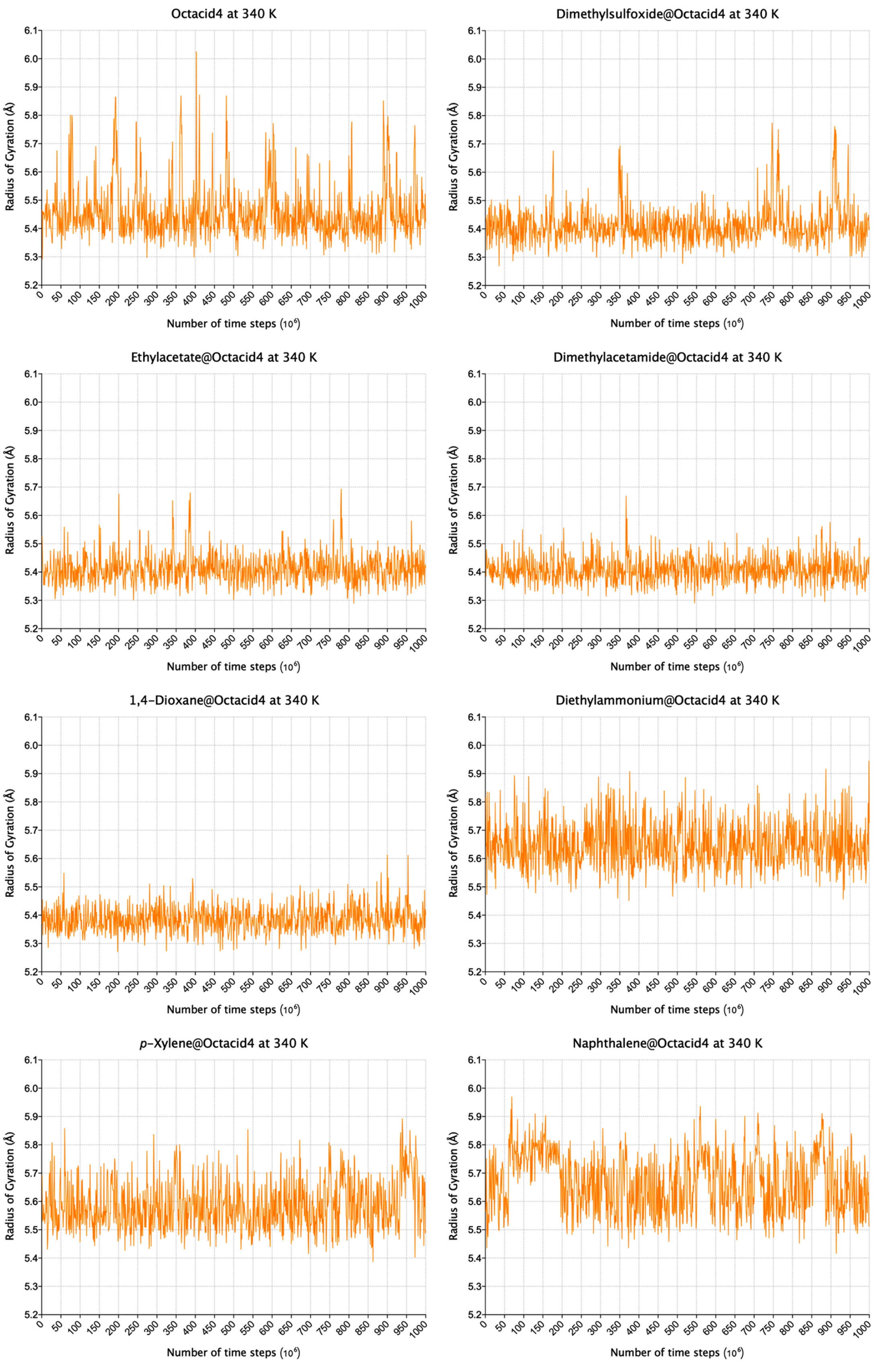

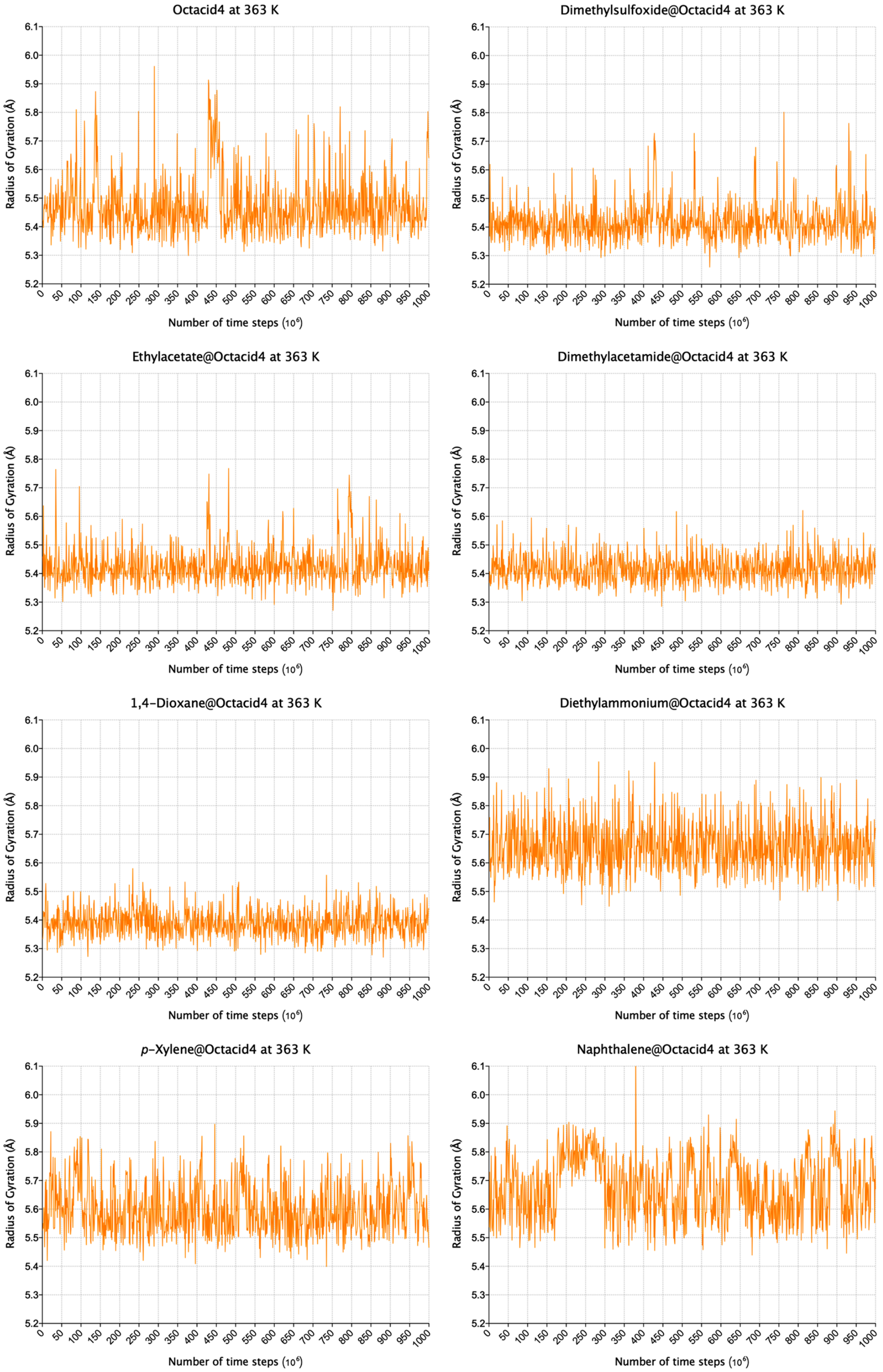

Fig. S3. Time series of radius of gyration of the Octacid4 cavity for the first of 20 distinct and independent MD simulations at 363 K.

Fig. S4. The most populated conformations of Octacid4 and its complexes in the MD simulations at 340 K. a Octacid4. **b** DMSO•Octacid4. **c** EtOAc•Octacid4. **d** DMA•Octacid4. **e** 1,4-Dioxane•Octacid4. **f** DEA•Octacid4. **g** *p*-Xylene•Octacid4. **h** Naphthalene•Octacid4. The representative and average conformations in the largest conformation cluster of a set of 20 MD simulations for each complex are shown in the left and right panels, respectively. No energy minimization was performed on these representative and average conformations. The sulfur, oxygen, nitrogen, and carbon atoms are in yellow, red, blue, and green, respectively. Hydrogen atoms, counter ions and water molecules are not displayed for clarity.

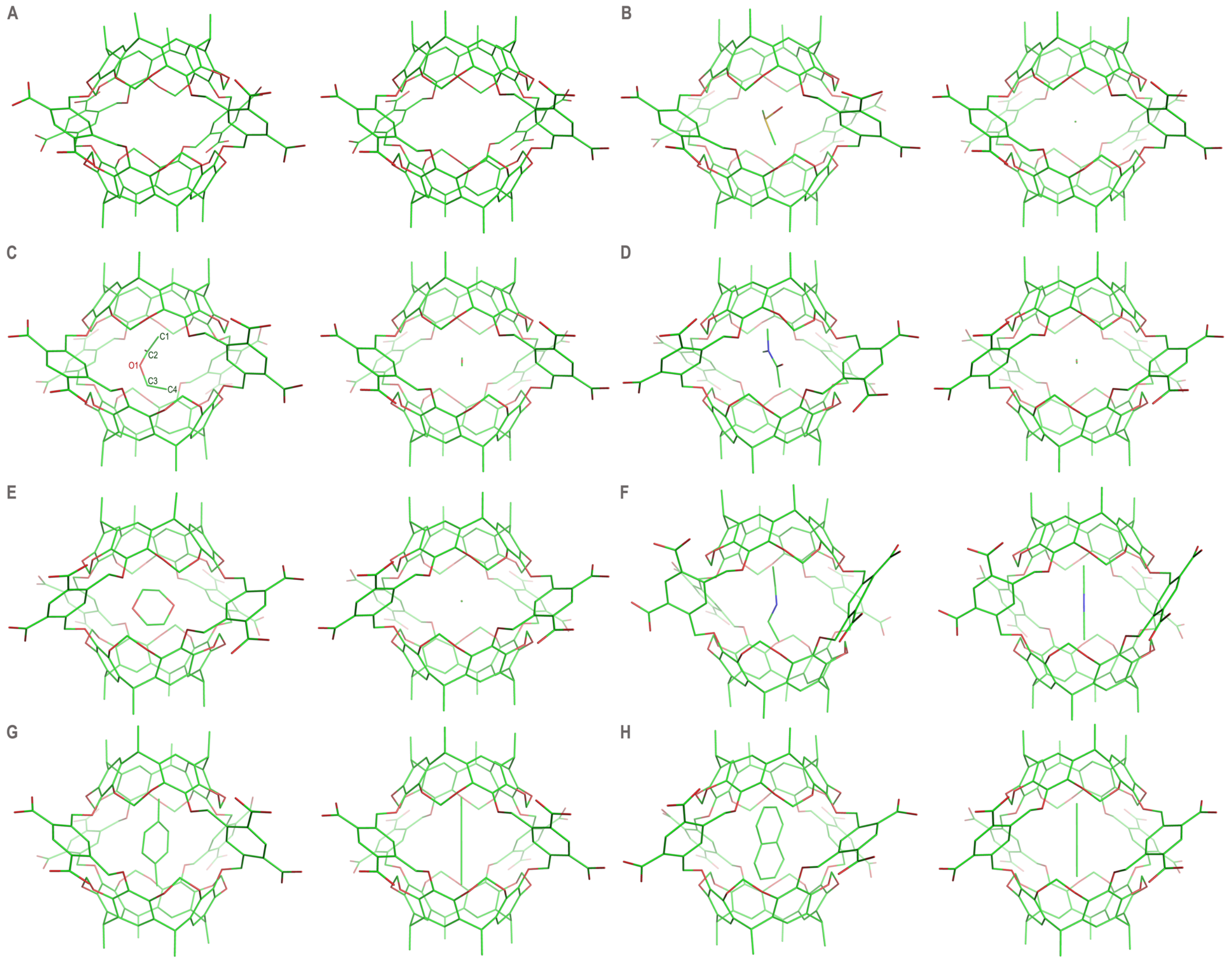

Fig. S5. The most populated conformations of Octacid4 and its complexes in the MD simulations at 363 K. a Octacid4. **b** DMSO•Octacid4. **c** EtOAc•Octacid4. **d** DMA•Octacid4. **e** 1,4-Dioxane•Octacid4. **f** DEA•Octacid4. **g** *p*-Xylene•Octacid4. **h** Naphthalene•Octacid4. The representative and average conformations in the largest conformation cluster of a set of 20 MD simulations for each complex are shown in the left and right panels, respectively. No energy minimization was performed on these representative and average conformations. The sulfur, oxygen, nitrogen, and carbon atoms are in yellow, red, blue, and green, respectively. Hydrogen atoms, counter ions and water molecules are not displayed for clarity.

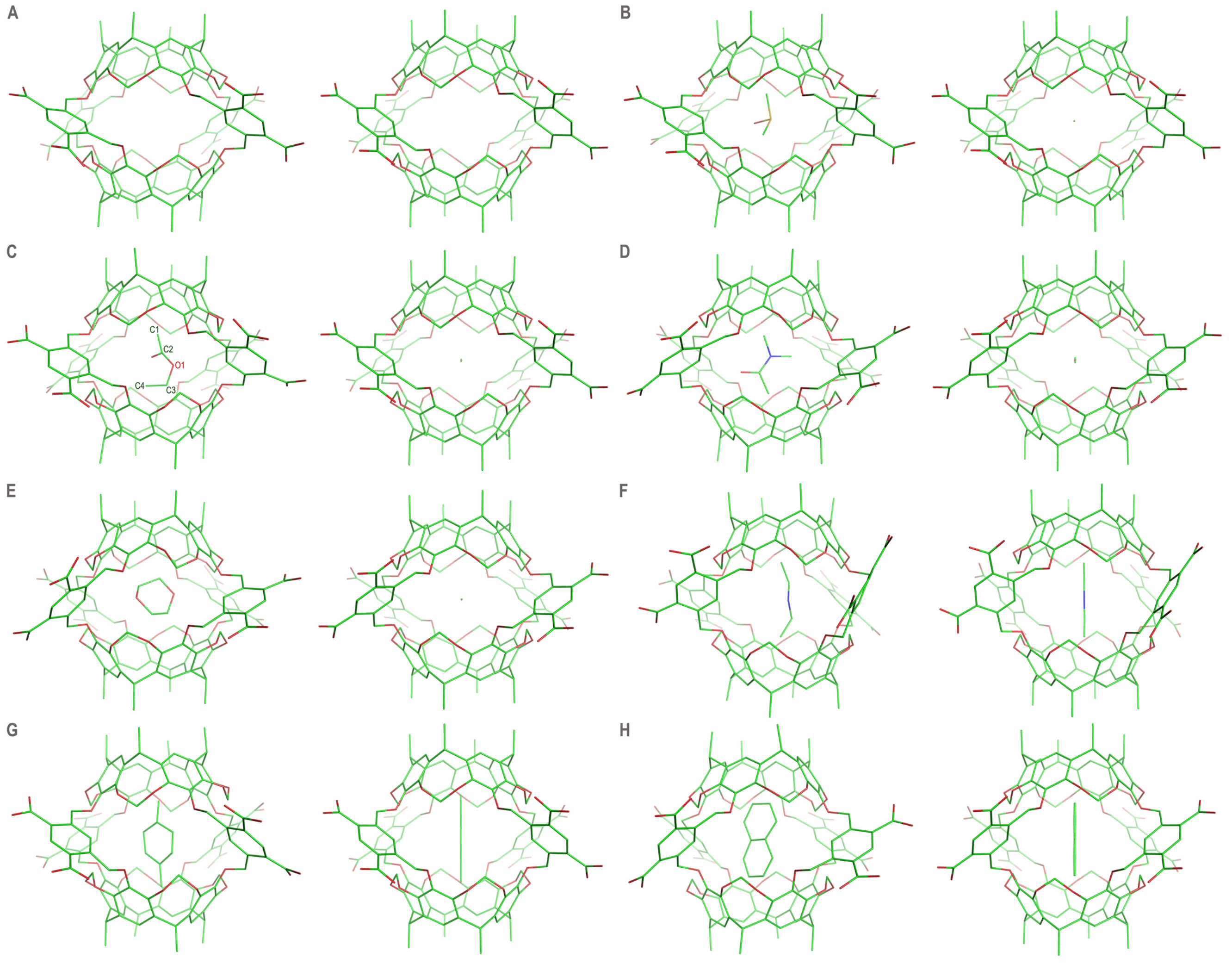

Supplement: Supplementary file 1 — Supplementary Information [file 42004_2021_469_MOESM1_ESM.pdf]
