# Supplementary material for: How the water-soluble hemicarcerand incarcerates guests at room temperature decoded with modular simulations
Source: Commun Chem. 2021 Mar 1;4:26. doi: 10.1038/s42004-021-00469-3 (PMC9814894; doi:10.1038/s42004-021-00469-3)
Supplement: Supplementary file 2 — Description of Additional Supplementary Files [file 42004_2021_469_MOESM2_ESM.pdf]

**Description of Additional Supplementary Files**

File Name: Supplementary Data 1
Description: The tLEaP input file for building Octacid4 using the AMBER HC1 library and frcmod files.

File Name: Supplementary Data 2
Description: The AMBER library and frcmod files for HC1, EtOAc, DMA, 1,4-dioxane, DEA, *p*-xylene, and naphthalene.

File Name: Supplementary Data 3
Description: The Cartesian coordinates for representative and average conformations of Octacid4 and its complexes each of which was derived from the largest conformation cluster of 20 316-ns MD simulations at 298 K, 340 K, and 363 K as well as the corresponding initial conformations for the simulations.
